# Prevalence and characteristics of diabetic foot ulcerations in Western Sydney

**DOI:** 10.1186/1757-1146-6-S1-O13

**Published:** 2013-05-31

**Authors:** Norafizah Haji Zaine, Kerry Hitos, John Fletcher, Mauro Vicaretti, Joshua Burns

**Affiliations:** 1 Arthritis and Musculoskeletal Research Group, The University of Sydney, Sydney, NSW, 2137, Australia; 2 Foot Wound Clinic, Department of Surgery, The University of Sydney, Westmead Hospital, NSW, 2145, Australia

## Background

Patients with diabetes are at high risk of developing foot ulcerations that can develop into non-healing wounds. Recent studies suggest that the lifetime risk of developing a diabetic foot ulcer is as high as 25%. The aim of this study was to determine the prevalence and characteristics of diabetic foot ulcerations (DFUs) at the Foot Wound Clinic at Westmead Hospital.

## Methods

In 2011, 318 patients were extracted for analysis from the Westmead Hospital Foot Wound Database on new diabetic foot ulcerations. Data on demographics, socio-economic, co-morbidities, foot ulcer characteristics and treatment were recorded on a standardised form adapted from the Eurodiale studies. Patients with Type 2 DM and Type 1 DM in outpatient clinics were included in the study.

## Results

In total, 74.5% of patients were diabetics. Demographics of diabetic foot ulcerations were: male (66.2%), mean age 67 years (range: 19-95 years), low socio-economic status (mean ABS postcode score 969, SD 119). DFU characteristics were: cross sectional area of 684.1mm^2^, volume of 6.3cm^3^, 33% on the forefoot, 67.9% acute and 12.3% chronic. The University of Texas (U/T) foot classification was category 6: the ischaemic limb (61.5%); category 4A: neuropathic wounds (34.6%) and others (3.9%). Predominant U/T wound types: 29.4% 1A and 12.8% 1C.

## Conclusion

Diabetic foot ulcers are prevalent in Western Sydney and are more likely to affect older males from a lower socioeconomic background. Understanding the other factors related to diabetic foot ulcers will assist the podiatrist in providing a more targeted management plan.

